# Reproducible hairy root transformation and spot-inoculation methods to study root symbioses of pea

**DOI:** 10.1186/1746-4811-7-46

**Published:** 2011-12-15

**Authors:** Scott R Clemow, Lindsey Clairmont, Lene H Madsen, Frédérique C Guinel

**Affiliations:** 1 Department of Biology, Wilfrid Laurier University, 75 University Avenue W., Waterloo, N2L 3C5, Ontario, Canada; 2 Department of Molecular Biology, Centre for Carbohydrate Recognition and Signalling, Aarhus University, Gustav Wields Vej 10, Aarhus C -8000 Denmark

**Keywords:** Composite plants, gene complementation, mycorrhizae, nodulation, pea mutants, targeted spot-inoculation, transformation

## Abstract

Pea has lagged behind other model legumes in the molecular study of nodulation and mycorrhizae-formation because of the difficulty to transform its roots and its poor growth on agar plates. Here we describe for pea 1) a transformation technique which permits the complementation of two known non-nodulating pea mutants, 2) a rhizobial inoculation method which allows the study of early cellular events giving rise to nodule primordia, and 3) a targeted fungal inoculation method which allows us to study short segments of mycorrhizal roots assured to be infected. These tools are certain to advance our knowledge of pea root symbioses.

## Background

*Pisum sativum *(pea) has been a model organism in plant research for more than a century; however, it has lagged behind other plants in becoming the model organism to study root symbioses, i.e., those resulting in nodulation and mycorrhiza-formation. Reasons for this stem from the fact that pea is a large plant which requires a large area for growth and has a relatively long life-cycle (~ 90 days). It has a large genome (about 4 × 10^9 ^base pairs [1]) still to be sequenced, and many molecular techniques, especially those of transformation, are difficult to use [1] to study the function of the genes involved in the nodulation process. Yet, the agricultural and economic importance of pea is significant, especially in Canada, so despite the difficulty in manipulating this species, it is relevant to study its beneficial root symbioses and to capitalize on its large collection of symbiotic mutants [2]. Pea production is expected to increase further as farming practises are focusing on agricultural sustainability, with farmers once again utilizing the benefits of crop rotation to decrease fertilizer applications.

Two legumes, *Lotus japonicus *and *Medicago truncatula *(barrel medic), have been used as model plants to study the plant-rhizobial and the plant-fungal relationships that lead to the fixation of nitrogen and the increased uptake of phosphorus, respectively [e.g., [3,4]]. These model organisms have helped advance our understanding of the molecular dialogue between plants, rhizobia, and fungi, and the signalling pathway which ensues. The early symbiotic events occurring in the root and leading to nodule organogenesis or to mycorrhiza formation have been a subject of great interest but are difficult to study [e.g., [5,6]]. Complications arise in locating in time and space specific root events, such as those that occur before the microsymbionts have entered the root and those that take place within non-translucent and thick roots after the microsymbiont entry [5,6]. Many techniques have been developed and refined to advance the study of symbioses in these model species.

The powerful technique of transformation has been widely used to study gene function and it has served as an important crop improvement tool [e.g., [7]]. Pea, like most other large legume plants, has been considered to be recalcitrant to transformation [8,9], although, as summarized in a recent paper [10], several successful methods have been developed by infecting explants with *Agrobacterium tumefaciens*. Unfortunately, most of these procedures are plagued by long shoot regeneration periods; indeed, up to 9 months [e.g., [11-13]] may be required before rooting can take place. Bean *et al. *[11] reported additional drawbacks including low fertility, phenotypic abnormalities, altered ploidy, and loss of transgene activity in subsequent generations. Despite the difficulties, one group has been successful at introducing in pea, via *A. tumefaciens*, a construct comprising the nodulation reporter *ENOD12A *gene [14]. The success rate was, however, low, and the transformed population not homogeneous as transformants varied in the number of gene copies inserted. The pitfalls associated with the above-mentioned methods make them labour-intensive and not efficient for research applications. If the gene of interest is involved in root biology, e.g., in rhizobial and mycorrhizal symbioses, a convenient way to avoid the problems encountered with *A. tumefaciens *transformation techniques is to use *Agrobacterium rhizogenes*, the agent responsible for hairy root disease. Pea is susceptible to this type of transformation, and isolated root-organ cultures have been obtained [15]. When studying nodulation, however, those cultures are not useful because nodule development requires shoot photosynthates [16]. To address this problem, techniques have been developed to create composite plants that have transformed roots but a wild-type shoot [17]. These chimeric plants have proven to be useful in studies of root nutrient uptake, hormone transport, and the nodulation and mycorrhizal symbiotic pathways [e.g., [7]]. Likely because of the difficulty encountered in pea transformation, there has been only one report of successful nodule formation on composite wild-type/transgenic pea plants [18], and gene complementation in pea mutants deficient in nodulation (i.e., Nod^- ^mutants) has not been previously reported. This has forced researchers studying pea to resort to the transformation of pea genes into close relatives, such as *Trifolium repens *(white clover) [19] or barrel medic [2,20].

Spot-inoculation is a technique that has been exploited to induce the formation of a nodule at a known location and has facilitated the identification of the first stages of nodule organogenesis, including pre-infection events [e.g., [5]]. This technique also enables one to know precisely the age of a developing or mature nodule, allowing a linkage between gene expression and a specific developmental stage. Spot-inoculation is now a common practice in *Lotus *and *Medicago *whereby plants are grown on agar plates with varying nutrients [e.g., [21,22]]. A similar technique has been employed in the study of mycorrhiza formation [23] and its adaptation for continuous observation with a confocal microscope has proven to be a powerful tool to gain insight into the events leading to root infection [6]. Yet spot-inoculation of larger plants such as pea has not been widely performed, likely because these are difficult to grow on agar plates.

Here, we present a protocol for the rapid development of transformed hairy roots on composite plants of *Pisum sativum *using *A. rhizogenes*. We show that the transformed roots of two non-nodulating pea mutants complemented with the appropriate wild-type gene are capable of nodulating with *Rhizobium leguminosarum*. Furthermore, we describe a technique of spot-inoculation refined for our species of interest, pea. We report that this technique is valuable for studying not only nodule organogenesis but also mycorrhizae. We trust that these techniques will be of great use to the many scientists worldwide interested in the study of pea root symbioses.

## Results

### Pea Transformation

#### Proof-of-concept

**Figure 1:**
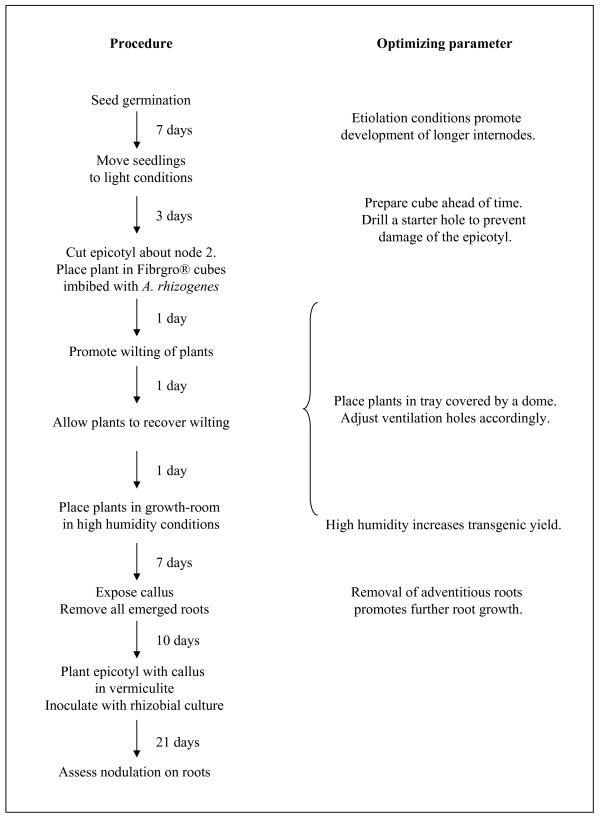
**Schematic representation of the time-line required for the generation of composite pea plants, with an emphasis on the parameters which must be optimized**.

**Figure 2:**
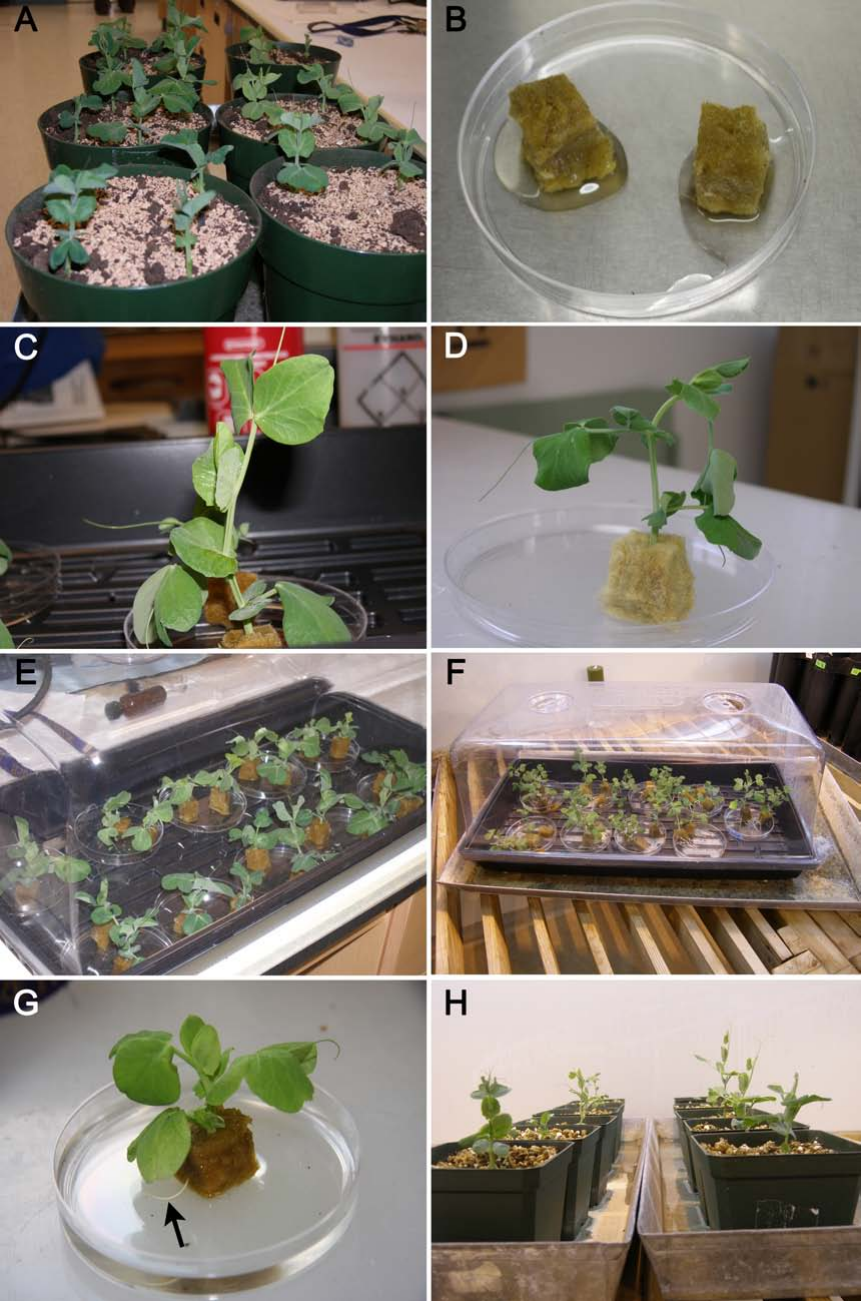
**Illustrations depicting the main steps for the production of composite pea plants**. **A**. Plants grown for 10 days in vermiculite (7 days in dark followed by 3 in light). **B**. Fibrgro^® ^cubes saturated with *A. rhizogenes*. **C**. A shoot of a 10 day-old plant cut above node 2 and inserted into Fibrgro^® ^cube. **D**. A wilted pea plant about to receive water for recovery. **E**. Plants placed in growth tray under a plastic dome. **F**. High humidity is provided by plastic lid with closed ventilation holes and metal tray with water. **G**. A root (arrow) protruding through a Fibrgro^® ^cube 10 days after *A. rhizogenes *infection. **H**. Callus-forming plants, the regenerated roots of which have been excised, placed in an upright position in vermiculite before being inoculated with *R. leguminosarum *3 days later.

**Figure 3:**
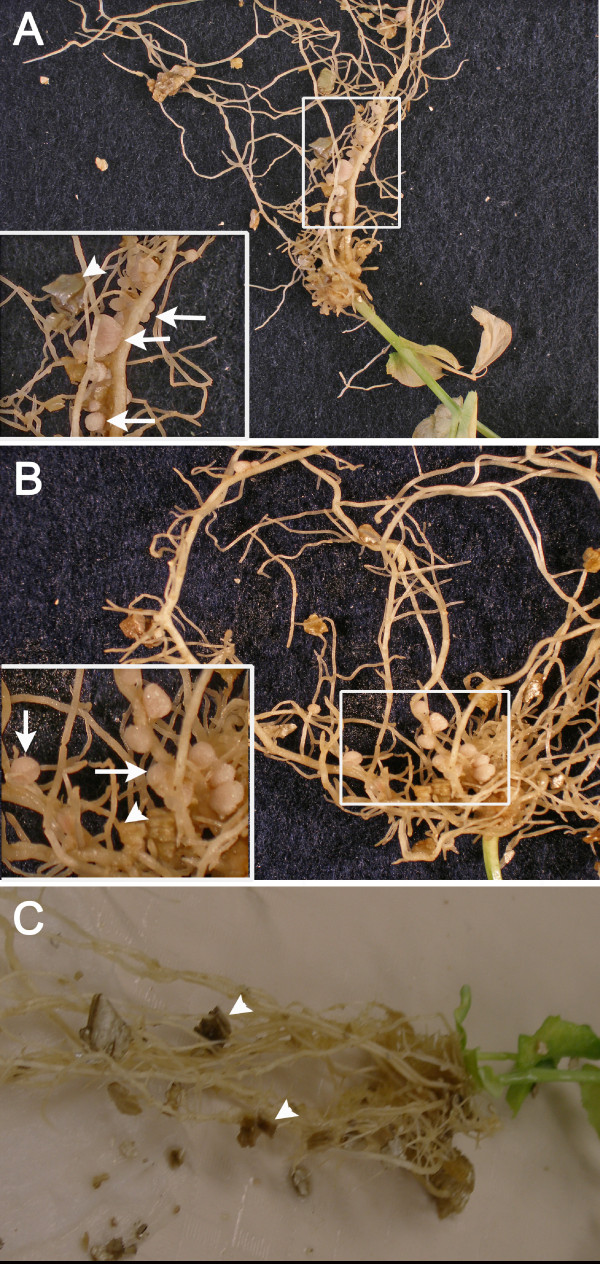
**Macro-photography of developing nodules on transformed roots**. Non-nodulation mutants P56 (**A**) and P5 (**B**) had their nodulation capabilities restored through *A. rhizogenes *transformation. Each inset shows a magnification of the area framed in the main photograph. Root nodules (arrows) that have emerged from the transformed roots should not be confused with vermiculite pieces (arrowheads). **C**. P56 mutant transformed with AR1193 lacking the *sym10 *construct did not exhibit any nodules.

In our hands, the best method to produce transformed composite pea plants (Figures 1 and 2) relied on the infection of 10 day-old pea seedlings using Fibrgro^® ^cubes imbibed with *A. rhizogenes *suspension as in Collier *et al. *[24]. After a short wilting period (Figure 2D), the treated plants were incubated in high humidity conditions (Figures 1 and 2F) until roots developed ~10 days after infection (Figure 2G). To assess the validity of our technique, we used Frisson and its mutants P5 [25] and P56 [26] as a tool to score for transformation efficiency. As described in [27], P5 and P56 are *sym10 *mutants that are defective in nod factor perception and thus are non-nodulating. Our rationale was that the complementation of these mutants with the wild-type *SYM10 *gene should reverse their nodulation phenotype and the presence of nodules should serve as the ultimate scoring for transformation success. *A. rhizogenes *strains AR12 and AR1193 [[17] and [28], respectively] carrying the *sym10 *genes were both used to transform the mutant plants. Using the described protocol (see Materials and methods, and Figures 1 and 2), we complemented successfully the *sym10 *mutants P5 and P56 because roots of composite plants challenged with *Rhizobium leguminosarum *had nodules 21 days after inoculation (Figure 3). Furthermore, no nodulation was observed on P5 or P56 mutant plants (n = 8 and 7, respectively) transformed with AR1193 carrying no construct (Figure 3), while nodulation occurred on wild type plants (n = 4) transformed with AR1193 (data not shown).

This high-throughput root transformation system is the first to show complementation of a nodulation gene in pea. We defined complementation efficiency as the ratio of *Agrobacterium*-infected plants which displayed at least one nodulated root (Table 1). In our experiments, strain AR12 was less efficient than strain AR1193 at producing transformed roots as less nodules were observed on the roots of plants transformed with AR12 (data not shown). To improve complementation efficiency using AR12, we applied antibiotic counter-selection to inhibit the growth of non-transformed roots in favor of transformed roots. A range of kanamycin concentrations (2 - 15 μg/mL) was tested but produced no satisfactory results, as treatments caused significant plant growth retardation. Because AR1193 was more efficient than AR12 at transforming pea, we pursued our optimization experiments using it solely.

**Table 1:** Success rate of *sym10 *complementation in pea mutants P5 and P56

Pea line	Treatment *	Number of plants	Number of plants with nodulated roots	Complementation efficiency
P5 (*sym10*)	Water	36	10	0.28
P5 (*sym10*)	IAA	21	4	0.19
P5 (*sym10*)	Roots excised	22	12	0.55
P56 (*sym10*)	Water	20	4	0.20
P56 (*sym10*)	IAA	14	5	0.36
P56 (*sym10*)	Roots excised	14	10	0.71
Latest round of transformation				
P5 (*sym10*)	Roots excised	22	16	0.73
P56 (*sym10*)	Roots excised	10	7	0.70

The excised shoots of P5 and P56 plants were exposed to *Agrobacterium rhizogenes *AR1193 carrying the *SYM10 *gene. Complementation efficiency represents the ratio of transformed plants with at least one root bearing nodules. With each passing round of transformation, adjustments were made to fine-tune the protocol. Thus the latest round is distinguished from the other trials to illustrate the outcome of the optimized protocol. * The different treatments were performed after the *Agrobacterium *inoculation in order to promote the formation of roots. The treatment where plants received only water until roots developed were considered as control. The plants undergoing the IAA treatment were given 10 μM IAA for 48 hours after transformation. The plants undergoing the root excision treatment were given water until roots developed; all roots protruding around the callus were excised before the callused epicotyls were transferred to pots.

#### Induction of root formation

To trigger the growth of transformed roots [29], we excised roots which had protruded from the callus (Figure 2G) as most of the roots that emerged were adventitious, i.e., arising from the stem. We also treated plants two days after AR1193 infection with IAA (0.1 - 10 μM) for 48 hours because IAA had been commonly used in rooting media [e.g., [30]]. To determine treatment impact, we compared treated plants to plants receiving only water (Table 1). Plants that received exogenous IAA had in general higher complementation efficiencies than plants receiving only water. The highest complementation efficiency, 70% for P56 and 73% for P5, was however obtained when all of the roots appearing through the Fibrgro^® ^cube were excised (Table 1). This treatment ultimately resulted in an average number of nodules per root of 9.6 and 7.3 for P5 and P56, respectively. The pruning seemingly promoted root growth from the callus while it limited further adventitious root development.

### Spot-inoculation

**Figure 4:**
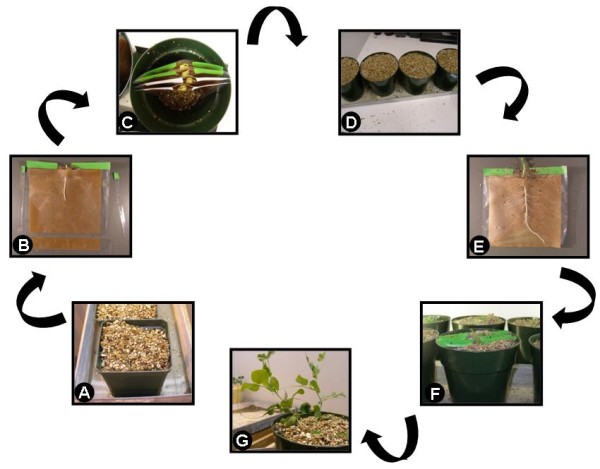
**Schematic representation of the important steps for spot-inoculation**. **A**. Imbibed seeds placed in vermiculite. **B**. Seedling radicle placed in trough of trimmed pouch. **C**. Pea cotyledons and epicotyls protruding from tightly-closed pouches. **D**. Pots, with pouches inside, filled with vermiculite to prevent light exposure. **E**. Inoculation 5 days later with *R. leguminosarum *bv. *vicieae *placed as a minute drop (seen as a black dot drawn with a marker on plastic) at the most susceptible zone of infection of several lateral roots. **F**. Pouches later returned to pots. **G**. Healthy plants allowed to grow until inspection.

**Figure 5:**
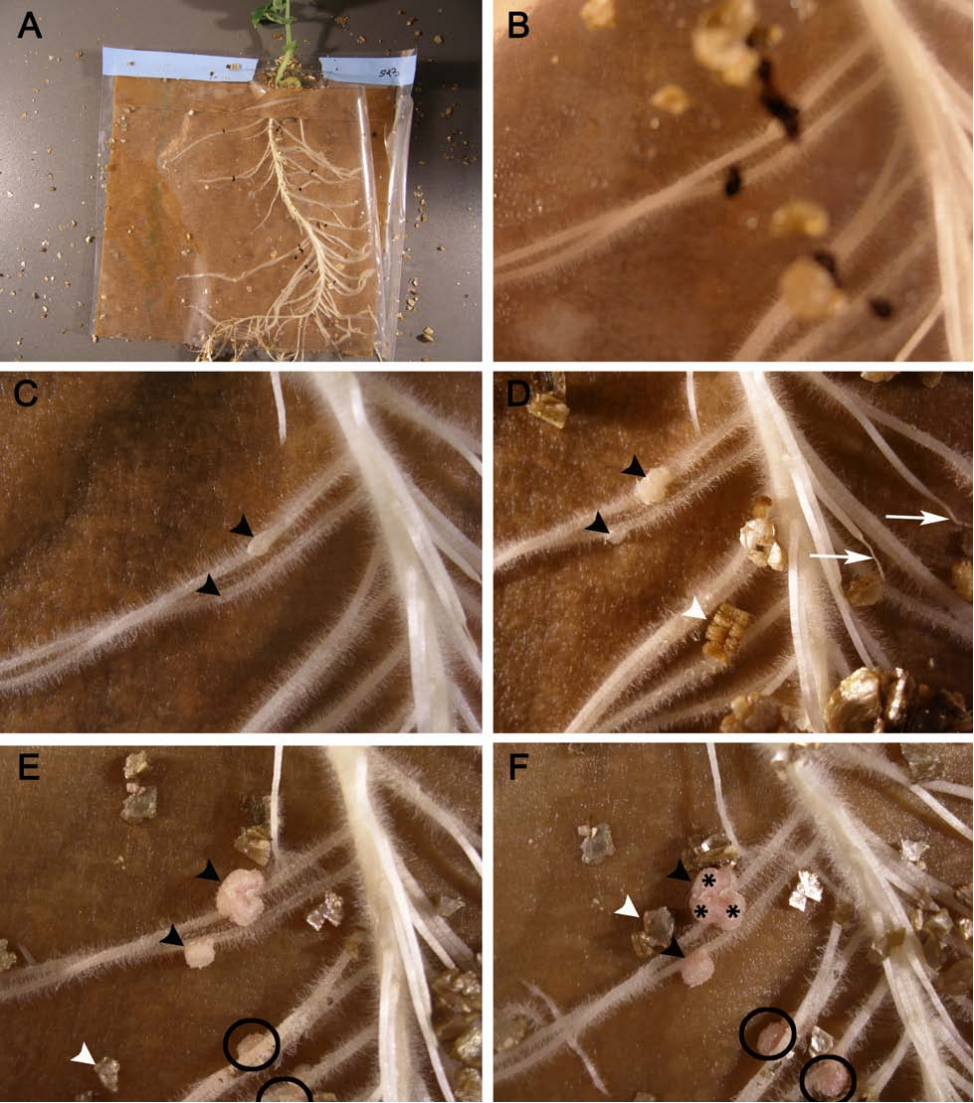
**Macro-photography of nodules developing on roots of plants grown in pouches**. **A**. An overview of an 8 day-old spot-inoculated plant. **B**. A close-up focussed on the root hairs of the seedling 5 DAI. The unfocussed black dots are located on the surface of the pouch and correspond to the different inoculation sites. In **C **to **F**, two nodules (arrowheads) can be seen developing over time. The nodules are five-day old (**C**), seven-day old (**D**), 10 day-old (**E**) and 14 day-old (**F**). In **E **and **F**, two additional nodules (black circles) appeared later at the spot-inoculation site; they cannot be given a specific age. One (asterisks in F) of the nodules appears much larger than the others; it is evident that this large nodule has multiple meristems. In **D**, two roots (arrows) which did not grow in contact with the filter paper of the pouch dried out. White arrowheads indicate pieces of vermiculite.

Spot-inoculation protocols have been developed for a variety of legumes but these techniques have not been easily transferred to pea. We developed a highly reliable rhizobial spot-inoculation technique by growing seedlings in growth pouches (see Materials and Methods) and placing a drop of the rhizobial inoculum on a lateral root located in the zone that is most susceptible to infection. We found that the substrate and the method of delivery of the inoculum were keys in the success of pea spot-inoculation. With the procedure presented in Material and Methods and in Figure 4, a nodule formed within ~0.5 cm from the intended location in 95-100% of the cases (Figure 5). Using this technique, it becomes possible to determine the exact age of the nodule from the time of inoculation to 42 days thereafter. We were able to apply what we learned with the rhizobial spot-inoculation and we targeted mycorrhizal infection.

#### Substrate Choice

Pea has a large root system, making it difficult to be cultivated over a long period of time on agar slides as Fåhraeus did with white clover seedlings [31] and impractical to use it for studying nodule development in mutants with delayed nodulation. Yet, a few researchers have been successful at spot-inoculating pea utilizing either the Fåhraeus technique [32] or agar-filled plates [33]. In our hands, unfortunately, healthy nodulating pea plants are not obtained on agar plates, and other labs have reported similar problems in the past [e.g., [34]]. With filter paper within a pouch as a substrate, the size of a plant is less of an issue for growth. Turgeon and Bauer [35] grew *Glycine max *(soybean) in germination pouches, held in an upright position in plastic trays with high walls, and spot-inoculated them with one to three nL drop of inoculum, which they placed in the root zone that is most susceptible to infection. In our attempts to replicate this technique for pea, the roots either tended to dry rapidly or became easily water-logged, thus stunting the growth of the plant and preventing the formation of nodules. To palliate this problem, we have cut the bottom part of the pouches which we then placed in pots filled with vermiculite (Figure 4B). The paper of the pouch is capable of acting as a wick and drawing water when needed from the wet vermiculite. Apparently, not all pouches are similar because recently a group of researchers obtained healthy-looking nodulated plants in such a system [36]. The paper in the pouch is, however, noticeably distinct from the one we used; the different result may also lie with the pea cultivars used, the method of pre-germination or the method of inoculation (flood-inoculation versus spot-inoculation) [36].

#### Rhizobial Inoculum Delivery

**Figure 6:**
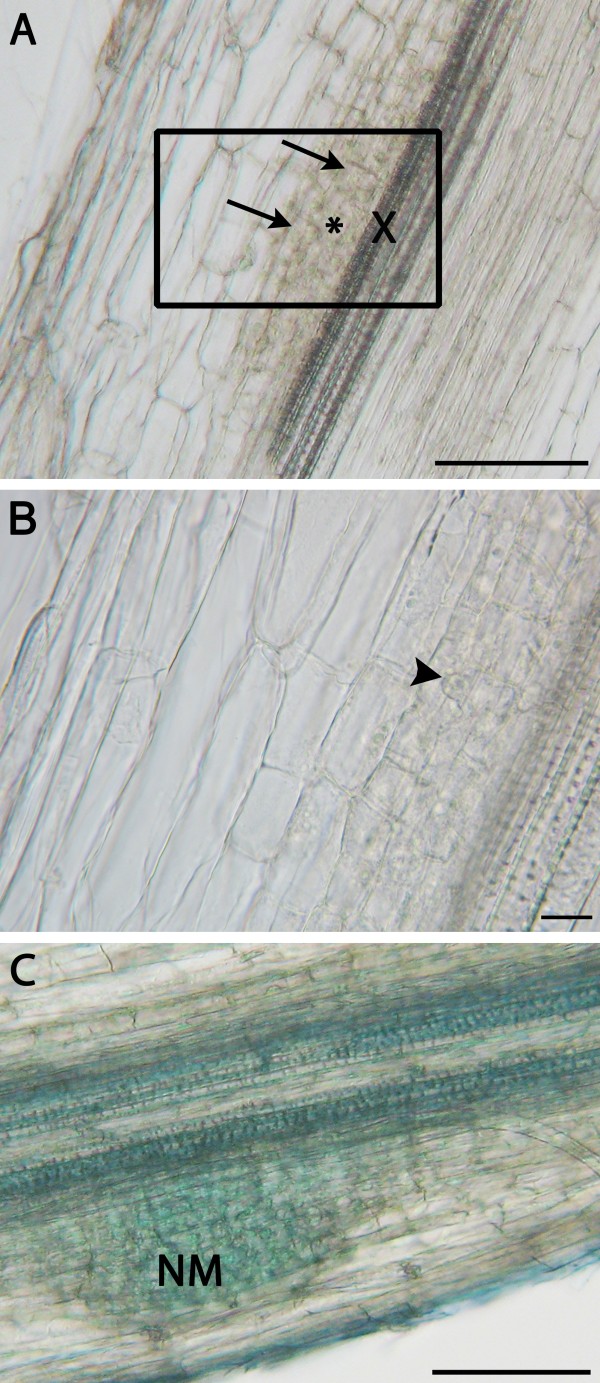
**Microscopy of spot-inoculated lateral roots**. **A**. An unstained longitudinal section of a spot-inoculated lateral root 24 hours after rhizobial inoculation showing anticlinal divisions in its inner cortex (arrows), and in its pericycle (asterisk), located adjacent to a xylem pole (X). Bar: 100 μm. **B**. A close-up on the area framed in Figure A. Metabolically active cells, one with a large nucleus (arrowhead), exhibit anticlinal divisions. Bar: 10 μm. **C**. A longitudinal section of a root inoculated 3 days earlier with rhizobia and stained with toluidine blue. The nodule meristem (NM) has passed the mid-point of the root cortical region. Bar: 100 μm. Note that for all the photographs, there was no indication of nodule emergence and it was only because of the spot-inoculation that these events were easily captured. All sections were made with a vibratome.

Diaz *et al. *[37] reported successful nodulation on the primary root of pea spot-inoculated with a drop of an inoculum made of a mixture of rhizobia and 0.15% agar. Employing this technique, van Brussel *et al. *[32] were in fact able to observe the first described pre-infection threads. However, when we tried this method, the agar-mixed rhizobia impeded root growth (drying the root in some cases) in the area of application and no nodules were observed. Therefore, we tested different means of delivering the inoculum. First, a small strip of Parafilm™ was used to hold the drop of inoculum in place at the desired location [38]. Second, a rhizobia-india ink suspension was applied [38]. Finally, glass micro-carriers which were successful in spot-inoculating alder with *Frankia *[39] were used to transfer rhizobia to the root and prevent the inoculum drop from moving away from the chosen location. The above techniques not only were tedious and time-consuming, but they failed to produce nodules locally. We found that two details were important to obtain nodules where we wanted them to develop (Figure 6). First, the bacterial micro-drop had to be delivered on a root system growing on a paper just damp; if the paper was too moist, the drop was quickly lost. Second, the drop should be left for a few seconds on the root before the pouch can be closed. Furthermore, roots which do not touch the paper of the pouch, or may not touch the paper once grown, should not be inoculated as they have an increased chance to dry out (Figure 5D).

#### Fungal Inoculum Delivery

**Figure 7:**
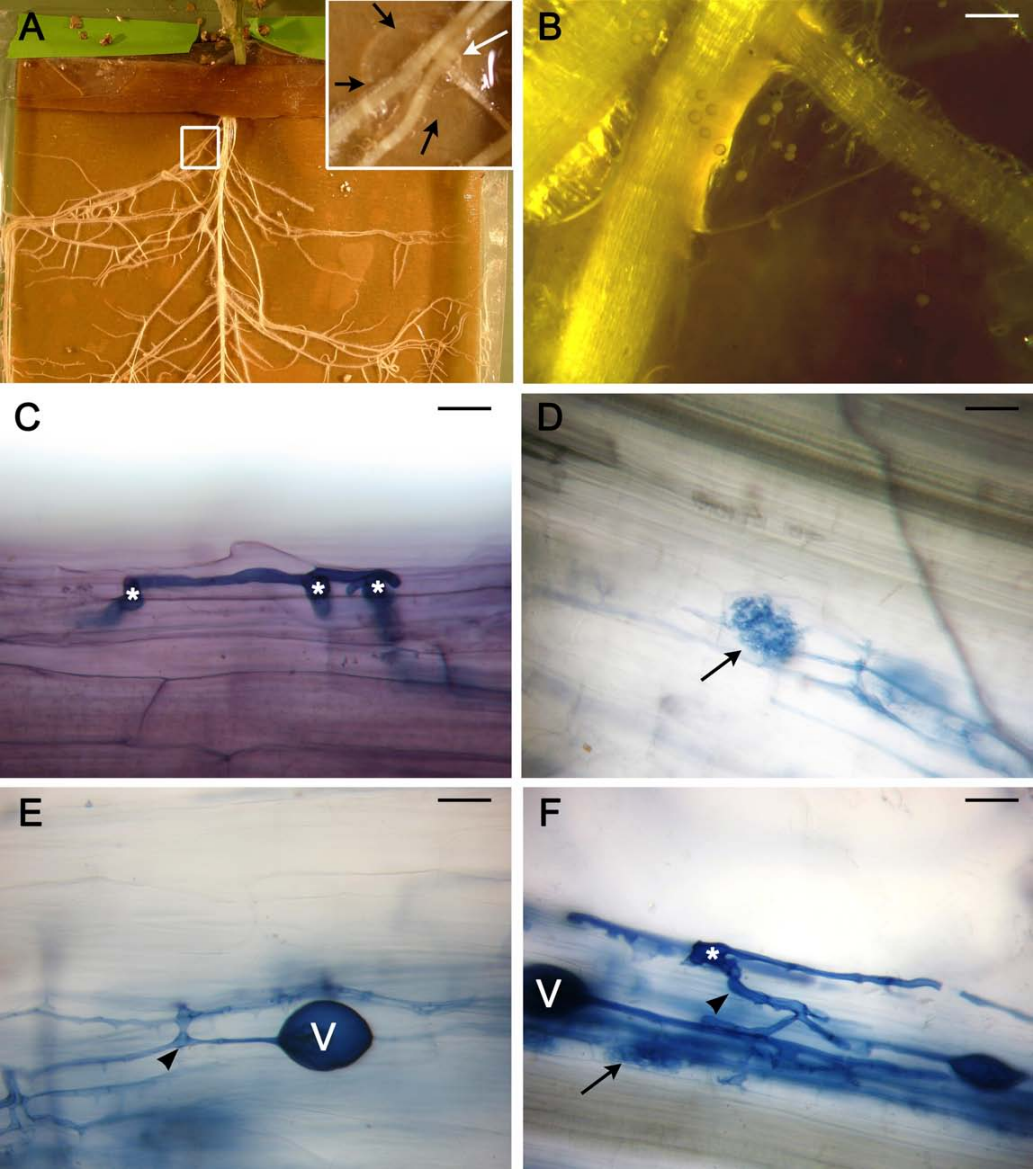
**Fungal spot-inoculation of plants grown in a pouch**. **A**. An overview of the root system of a 40-day old plant grown in a pouch. The plant was challenged when it was 5 day-old with fungal spores located in an agar plug. Inset: A close-up of the area indicated by a white square in A. The plug (delineated by arrows) was placed below the lateral roots. **B**. Close up on the roots which were pushed gently into the plug containing the fungal spores. Bar: 0.5 mm. **C**. An extra-radical hypha and some hyphopodia (asterisks) on the surface of a cleared root inoculated 21 days earlier. **D**. Arbuscules located within inner cortical cells of a cleared root inoculated 35 days earlier. **E**. Intraradical hyphae and a vesicle in the cortex of a root 35 DAI. **F**. An infection unit with extensive hyphal branches; note the hyphopodium (asterisk), the main intraradical hypha (arrowhead), an arbuscule (arrow), and a vesicle (V). C - F: Bar: 20 μm; ink-vinegar staining.

To investigate mycorrhizal infection in pea, we adapted the method of delivery used in the rhizobial spot-inoculation, i.e., we brought a small amount of fungal propagules to plants grown in pouches. Fungal spores obtained from root-organ cultures [40] were transferred to the plant within an agar plug (Figure 7A, inset, and  7B). The plug, adhering to the paper of the pouch with melted agar, was placed below a growing lateral root. Root segments expected to be infected were excised at 21 and 35 days after inoculation and stained to visualize infection. Three of the eight plants harvested and processed at 21 days after inoculation (DAI) showed evidence of fungal infection, including extraradical hyphae and hyphopodia (Figure 7C), at the spot-inoculation site. At 35 DAI, success was rated at 78%, i.e., 11 infected roots out of 14 displayed infection units, which were comprised of intraradical hyphae, arbuscules (Figure 7D), or vesicles (Figure 7E).

## Discussion

Here we focus on the wide applications of the three techniques presented in this paper. *A. rhizogenes *root transformation of legumes once thought to be recalcitrant, such as soybean [e.g., [29]], *Phaseolus vulgaris *L. [41], *Aeschynomene indica *[42], and now pea, should bring new insight into the roles of genes involved in nodulation and mycorrhizae formation. Model legumes have opened the avenue to molecular studies on root symbioses and to transfer the acquired knowledge to transformation and molecular understanding of the pulses is essential. With the transformation technique described here, the number of nodulated roots per pea plant was between 1 and 4, with average complementation efficiency (at least one nodulated root per plant) of 0.70. The transformation efficiency with our system should be at least as high as the observed complementation efficiency because it is likely that some transformed roots did not nodulate. These numbers should be compared to those obtained by Collier *et al. *[24] for soybean who reported between 2 and 4 transformed roots per transformed plants with a transformation efficiency of ~ 80%. It is of interest to note that although not equivalent in their efficiency, both agrobacterial strains used (AR12 and AR1193) were successful in transforming pea. Future directions should include a reliable marker, such as pHairyRed [43], for an easy and convenient selection of transformed roots.

The spot-inoculation technique, whether rhizobial or fungal, should be useful in a number of different studies. The infected roots can be cut 0.5 cm on either side of the spot at any time after inoculation and sectioned (fresh or fixed) or cleared to study specific ages of nodules (Figure 6) or mycorrhizae (Figure 7), respectively. These anatomical studies may be complemented with molecular studies whereby gene expression is assessed in response to microbial challenges. With mock-inoculated roots used as a control, expression of specific genes can be correlated to specific symbiotic events (Clemow and Guinel, unpublished). Furthermore, at all times, the right side of the plastic pouch can be cut so that the plastic cover becomes a flap which can be lifted and replaced at the researcher's convenience. This permits to follow the infection, especially that resulting in nodulation, over time by macro-photography (Figure 5), so that the dynamic growth of lateral roots with their nodules can be followed. In the case where rhizobia transformed with either a lacZ gene or another visual marker such as green fluorescent protein are used to perform spot-inoculation, rhizobial progression can be tracked.

We see some great advantages for our spot-inoculation methods. For the rhizobial delivery, in contrast to prior techniques which have relied on the primary root of pea for spot-inoculation [37], our method utilizes its lateral roots which are where this species develops the majority of its nodules [44]. The fact that pea primary and lateral roots have different anatomy [45] could have an unforeseen effect on nodule development. For both symbioses, in most cases, spot-inoculation guarantees infection events close to the spot. Needless to say, this shortens tremendously the time needed to look for symbiotic structures. The technique not only allows for early detection of events but also permits an easier characterization of nodulation (Macdonald and Guinel, unpublished) and mycorrhizae-formation mutants.

One minor inconvenience with our technique is that it does not limit nodule production to one specific location as nodules occasionally developed elsewhere on the root system where rhizobia were not applied. However, since we are interested in knowing the precise age of a developing/nodule, we do not take these "straggling" nodules into account. Also, occasionally spot-inoculated roots develop more than one nodule in the inoculated area.

## Conclusions

The techniques described here should open many doors to researchers working on pea root symbioses. Root transformation will allow not only nodulation gene complementation in pea but also the alteration of expression of specific genes of interest. Furthermore, the spot-inoculation technique should increase greatly the probability of finding infection events; it will especially permit the study of pre-infection events in the roots of plants grown in pouches challenged with either rhizobia or mycorrhizal fungi.

## Materials and methods

### Plant materials, bacterial strains and vectors, and fungal inoculum

Seeds of *Pisum sativum *L. cv. Sparkle and cv. Frisson, and seeds of its mutants P5 and P56 [generous gift from Gérard Duc (Dijon, France)], were surface-sterilized, imbibed, and planted in sterile vermiculite to germinate (see below for specifics). Unless otherwise mentioned, *P. sativum *plants were grown under incandescent (OGE 600 hour, 60 - 120 W, 120 Volts, General Electric) and cool white fluorescent lights (Watt-Miser GE, F96TIZ-CW-HO-WM) in a growth-room with a 23°C/18°C, 16 h/8 h light/dark regime. The total light intensity received by the plants was 280 μmol·m^-2^·s^-1^.

*Agrobacterium rhizogenes *AR12 and AR1193 [[17] and [28], respectively] containing *SYM10 *[27] were cultured in 100 mL of Luria Bertani (LB) broth with 100 μg/mL rifampicin and 100 μg/mL spectinomycin (all from Bioshop, Burlington, ON). As a control, AR1193 with no *SYM10 *construct was grown in LB with 100 μg/mL rifampicin. Cultures were grown at 28°C for 24 hours on an orbital shaker (New Brunswick Scientific, Edison, NJ) at 200 rpm until the culture reached an absorbance between 0.6 and 0.8 at 600 nm on a spectrophotometer (Varian Inc., Mississauga, ON). The bacterial culture was then transferred to sterile centrifuge tubes and spun at 6000 × g for 15 min, 4°C. The pellet was re-suspended in ice-cold, 1/4 × Murashige and Skoog basal medium (Sigma-Aldrich M5524, Oakville, ON), pH 5.8, until an optical density of 0.3 at 600 nm was reached.

*Rhizobium leguminosarum *bv. *viciae *128C53K (gift from Dr. S. Smith, Milwaukee, WI) was kept on slants at -20°C and cultured in 20 mL of yeast-mannitol broth, consisting of (g/L) mannitol 10.0, K_2_HPO_4 _0.5, MgSO_4 _0.2, NaCl 0.1, yeast extract 0.4 and of pH 6.8. The medium inoculated with a loop-full of stock culture was grown at 25°C in Erlenmeyer flasks on an orbital shaker at 100 rpm. Cells were grown to late log phase (48 hours) at which time the viable cell density was 1.2 × 10^6 ^colony forming units (CFUs)/mL.

Phytagel™media plugs containing spores of the AM fungus *Glomus irregulare *(DAOM 197919) were obtained from Agriculture and Agri-Food Canada's Glomeromycota in vitro collection. The fungus was propagated using root-organ cultures [40] of transformed carrot (*Daucus carota*) roots (Culture Collection, Mycothéque de l'Université Catholique de Louvain, Belgium).

### Pea transformation

Figures 1 and 2 highlight the important steps of the protocol with which we obtained the highest transformation efficiencies.

1| Surface-sterilize seeds in 8% bleach for 5 minutes to eliminate microbial and fungal contamination during germination. Wash seeds in three rinses of sterile water for 1 minute each and store them in sterile water in darkness for 12 to 15 hours to imbibe.

2| Plant in vermiculite imbibed seeds at a depth of 1-2 cm and let grow in the dark until the seedlings are ~ 8 cm tall [about 7 days after planting (DAP)].

NOTE: The dark treatment promotes etiolation and thus facilitates the shoot excision at a later step.

3| Set plants into a controlled growth-room for 3 days to provide time for the leaves to develop (Figure 2A).

4| Cut Fibrgro^® ^cubes (Homegrown Hydroponics Inc., Breslau, ON) into smaller cubes (~ 2.5 cm^3^). Use a dissecting probe to make a small starter hole, a quarter to half way through the cube, into which the stem of the plant will be inserted (step 7). Sterilize the cubes (pre-vacuum cycle for 20 min) in autoclave. 

NOTE: Without this hole the stems of the plants broke easily when inserted.

5| Prepare the culture of *A. rhizogenes *9 DAP as described above.

6| Place 2 to 4 sterile Fibrgro^® ^cubes, with the holes facing up, in 12 cm sterile Petri plates. Inoculate the cubes with the diluted *A. rhizogenes *culture until complete saturation; 4 to 7 mL of inoculum is usually sufficient per cube (Figure 2B).

7| Cut off the shoot of 10-day old plants above node 2 with a clean razor blade and place their stem gently in the hole of the Fibrgro^® ^cube (Figure 2C).

NOTE: When the distance between node 2 and 3 is too short, the plant is cut above node 1 instead so that the leaves are never in contact with the inoculated cube.

8| Place the Petri plates containing the plant/cubes in a plastic tray with drainage holes and cover with a transparent dome with ventilation holes closed (Homegrown Hydroponics Inc., Breslau, ON). Leave tray on lab bench for 24 hours (temperature of 21°C and light intensity of about 10 μmol·m^-2^·s^-1^). Remove the lid on the following day and leave the plants on the bench for an additional 24 hours to promote wilting of the shoots (Figure 2D).

NOTE: The wilting period is required for a callus to develop as it likely draws the inoculum into the plant [24]).

9| Add sterile water to the Petri plates when the stems are wilted, and replace the dome for an additional 24 hours (Figure 2E). Place the plastic tray into a metal watering tray and set the plants in the growth room (Figure 2F). Add water to the metal tray to cover the bottom of the plastic tray.

NOTE: Keep the plastic dome snug to the plastic tray and the ventilation holes closed to enhance humidity, which increases transgenic yield [29].

10| Dissect carefully with a pair of tweezers the Fibrgro^® ^cube away from the roots when the roots are visible (~10DAI) (Figure 2G). Excise all roots with a sterile blade.

NOTE: At this stage most of the roots are adventitious and their removal promotes root growth from the callus.

11| Transfer the callus-forming shoot with its epicotyl to a pot filled with sterile vermiculite; tightly pack the vermiculite around the rootless callus so that the shoot is kept upright (Figure 2H).

12| Two days before inoculation, grow a culture of *R. leguminosarum *as explained above. Inoculate the plants with 5 mL of a 5% bacterial solution 3 days after moving them to vermiculite.

13| Inspect plants 21 DAI and check for nodules.

### Rhizobial Spot-Inoculation of Pea

Figure 4 highlights the important steps in the protocol for successful spot-inoculation.

1| As 1| above.

2| Plant seeds in 7.5 cm pots filled with sterile vermiculite and let grow for 3 days (Figure 4A).

NOTE: Growing seeds in vermiculite is crucial to obtain plants with straight roots for easy transfer to pouches. It is important to allow the pea seedlings to develop for 3 days as the length of the radicle is optimal at this time.

3| Three days later, prepare to transfer one seedling per germination pouch (cyg™ seed germination pouch, Mega International, St-Paul, MN). Make a hole with a clean razor blade in the top centre of the pouch filter paper, so that the root enters the pouch but the cotyledons do not fall through the hole.

4| Wet the pouch with sterile low-nitrogen nutrient solution [as in [44]] until the entire surface of the filter paper has absorbed liquid (~ 7 mL).

NOTE: Micro-nutrients such as boron are required for nodules to develop [46]. It is essential to supply these nutrients instead of water alone as the seedling will not have access to them in a soil-free environment.

5| Remove gently the seedlings from the pots, and the seed-coat from the seedling, and place the primary root through the hole previously made (Figure 4B). The root must be in contact with the moist filter paper of the growth pouch.

6| Tape the top of the pouch closed, leaving a hole for the shoot to emerge (Figure 4B). Taping the pouch helps to maintain a moist inner environment preventing the roots from drying out. It also prevents vermiculite from entering the pouch (see below).

7| Cut a piece of the growth pouch 2.5 cm from its bottom with a pair of scissors (Figure 4B). The filter paper within the pouch will thus be in contact with the moist vermiculite (see below); this will ensure a constant supply of water to the developing roots while at the same time prevent any water-logging. The sides of the pouch can be trimmed for a better fitting when the pouches are inserted into the pots.

8| Place the growth pouches with the seedlings in 15 cm diameter pots (Figure 4C) and fill those with sterile vermiculite to cover completely the pouches (Figure 4D).

NOTE: Covering the pouch with vermiculite prevents light from reaching the roots; this is important as exposure to light inhibits pea nodulation [47].

9| Moisten the vermiculite with water and place the pots in trays in a controlled growth-room. Water the plants by adding water to the tray. The vermiculite maintains a humid environment for the pouch and filter paper.

10| Two days before inoculation, grow a rhizobial culture as above.

NOTE: Do not water the plants during this time to reduce the amount of residual water in the pouch. If the pouches are too wet, the inoculum drop will not remain on the root. It is of prime importance to time the earlier watering so that at this stage the pouches are only damp.

11| After 5 days of growth in the pouches, lateral roots will be of an optimal length for spot-inoculation (Figure 4E). Remove the pouches from the pots and find under the dissecting microscope the zone that is the most susceptible to infection, i.e., the region where roots hairs are starting to emerge from the root. This location is usually 0.5-1 cm away from the root tip.

NOTE: Condensation on the inside of the plastic pouch can impede the viewing of roots. Gently lift the top layer of the pouch from the bottom and use a sterile filter paper to remove the water droplets.

12| Mark the zone of the most susceptibility on the outside of the pouch with a water-proof marker (Figure 4E). Do not damage the root when applying the marker; if needed, hold the top surface of the pouch away from the root and apply the mark to the desired location.

13| Lift the top plastic sheet at the bottom end of the pouch and apply with a micro-pipette onto the root a 0.5 μL drop of rhizobial suspension adjusted to the viable cell density of 6.4 × 10^4 ^CFUs/mL.

NOTE: Do not place the top of the pouch back onto the root right away; let the drop sink in the root and paper before closing the pouch.

14| Return the pouches to the pots and cover with vermiculite (Figure 4F). Water the plants when needed; a good indication to do so is given when the colour of the pouch changes as it starts to dry. Harvest at the desired time (Figure 4G).

15| In our experiments carried out under the described conditions, we observed nodulation events, such as divisions of pericycle cells, as early as 24 hours after spot-inoculation; pink nodules are seen at 10 DAI.

### Fungal Spot-Inoculation of Pea

The protocol used in the fungal spot inoculation of pea begins with steps 1-9 of the rhizobial spot-inoculation protocol as stated above, except for step 4 where a low-phosphate nutrient solution [as in [48]] is used instead of a low-nitrogen one.

10| After 5 days of growth in pouches, prepare enough freshly autoclaved Phytagel™ (Sigma P8961) medium [0.3% in minimal medium [23,49]] so that there is approximately 1 mL of liquid per plant to be inoculated.

NOTE: The medium should be kept warm (60°C) to maintain its liquid form until the time of inoculation.

11| Remove each pouch from the pots; under axenic conditions, choose a location along one or more lateral roots for inoculation to occur. Mark this location on the top of the plastic sheet with a waterproof marker.

12| Lift the top plastic sheet at the bottom end and gently lift the lateral root chosen using tweezers. Apply 1 mL of liquid Phytagel™ to the filter paper of the growth pouch in the desired location below the selected lateral root. Using another set of tweezers, place a media plug containing the fungal inoculum onto the Phytagel™ and lower the root into place over the top of the plug, gently pushing the root into the medium of the plug.

13| Allow a minute or so for the plug to set in place and for the Phytagel-™ to solidify before lowering the plastic sheet. Return the pouches to the pots and cover them with fresh vermiculite. Water the plants when needed; however, be mindful of overwatering which can encourage fungal contamination of the pouches. Harvest plants at the desired time.

14| When harvesting the plants, remove the pouch from pots with special care so that none of the roots within the pouch are disturbed. Note the location of the plug and make cuts in the plastic to expose the root system. Remove the fragment of lateral root that was placed on top of the plug using a scalpel blade. Clear the root fragment and stain it with ink-vinegar [50] in order to observe fungal infection.

## List of Abbreviations

CFU: colony forming unit; DAI: day after inoculation; DAP: day after planting; IAA: indole-3-acetic acid; LB: Luria Bertoni; rpm: rotations per minute.

## Competing interests

The authors declare that they have no competing interests.

## Authors' contributions

SRC conceived, designed, and carried out the experiments of transformation and rhizobial spot-inoculation. LC conceived and performed the experiments of fungal inoculation. LHM made the *SYM10 *construct and discussed experimental approaches with SRC. SRC wrote the first draft of the manuscript. FCG directed the experiments and wrote the final draft of this manuscript. All authors have read and approved the final manuscript.
